# Trichodermin Induces G0/G1 Cell Cycle Arrest by Inhibiting c-Myc in Ovarian Cancer Cells and Tumor Xenograft-Bearing Mice

**DOI:** 10.3390/ijms22095022

**Published:** 2021-05-09

**Authors:** Ying Gao, Sarah L. Miles, Piyali Dasgupta, Gary O. Rankin, Stephen Cutler, Yi Charlie Chen

**Affiliations:** 1 Tea Research Institute, Chinese Academy of Agricultural Sciences, Hangzhou 310008, China; yinggao@tricaas.com; 2 Department of Biomedical Sciences, Joan C. Edwards School of Medicine, Marshall University, Huntington, WV 25755, USA; kittlaus1@marshall.edu (S.L.M.); dasgupta@marshall.edu (P.D.); rankin@marshall.edu (G.O.R.); 3 College of Pharmacy, University of South Carolina, CLS Columbia, Columbia, SC 29208, USA; sjcutler@cop.sc.edu; 4 College of Health, Science, Technology and Mathematics, Alderson Broaddus University, Philippi, WV 26416, USA

**Keywords:** trichodermin, A2780/CP70 cells, OVCAR-3 cells, c-Myc, cell cycle arrest, Cdc25A

## Abstract

Ovarian cancer is a fatal gynecological cancer because of a lack of early diagnosis, which often relapses as chemoresistant. Trichodermin, a trichothecene first isolated from *Trichoderma viride*, is an inhibitor of eukaryotic protein synthesis. However, whether trichodermin is able to suppress ovarian cancer or not was unclear. In this study, trichodermin (0.5 µM or greater) significantly decreased the proliferation of two ovarian cancer cell lines A2780/CP70 and OVCAR-3. Normal ovarian IOSE 346 cells were much less susceptible to trichodermin than the cancer cell lines. Trichodermin predominantly inhibited ovarian cancer cells by inducing G0/G1 cell cycle arrest rather than apoptosis. Trichodermin decreased the expression of cyclin D1, CDK4, CDK2, retinoblastoma protein, Cdc25A, and c-Myc but showed little effect on the expression of p21^Waf1/Cip1^, p27^Kip1^, or p16^Ink4a^. c-Myc was a key target of trichodermin. Trichodermin regulated the expression of Cdc25A and its downstream proteins via c-Myc. Overexpression of c-Myc attenuated trichodermin’s anti-ovarian cancer activity. In addition, trichodermin decelerated tumor growth in BALB/c nude mice, proving its effectiveness in vivo. These findings suggested that trichodermin has the potential to contribute to the treatment of ovarian cancer.

## 1. Introduction

Ovarian cancer has one of the highest fatality rates among gynecological cancers. The estimated number of new ovarian cancer cases and associated mortalities in the United States in 2021 are 21,410 and 13,770, respectively [1]. Though the incidence of ovarian cancer is not as frequent as other cancers, its prognosis is very poor [1]. Lacking early detection and effective treatment are related to the lethality of ovarian cancer [1]. Developing novel methods for the early diagnosis of ovarian cancer and exploring effective therapeutic treatments are two main strategies to decrease mortality. At present, the combination of taxol- and platinum-based chemotherapy is the standard first-line treatment for ovarian cancer after cytoreductive surgery [2]. Most recently, monoclonal antibodies or small molecules which inhibit vascular endothelial growth factor (e.g., Bevacizumab) or poly ADP-ribose polymerase (e.g., Olaparib, Rucaparib, and Niraparib) are approved by the US Food and Drug Administration for recurrent or advanced ovarian cancer treatment as well as maintenance therapy [3]. Nevertheless, both traditional chemotherapeutic agents and novel targeted drugs have drawbacks. A significant proportion of ovarian cancer patients do not respond to traditional chemotherapeutic agents [4]. Some ovarian cancer patients develop resistance after receiving several rounds of traditional chemotherapy [5]. When ovarian cancer relapses, it often turns out to be chemo-insensitive [5]. In addition, the side effects of traditional chemotherapy are severe and extensive. Targeted drugs are less toxic than traditional chemotherapeutic agents, but they still have side effects, such as hypertension and skin problems [3]. Sadly, evidence suggests that certain side effects of targeted therapy are linked to outcomes [6]. In addition, the cost of targeted drugs is high. Therefore, scientists are still working hard on discovering or designing compounds with high efficiency, few adverse effects, and a relatively low price.

**Figure 1:**
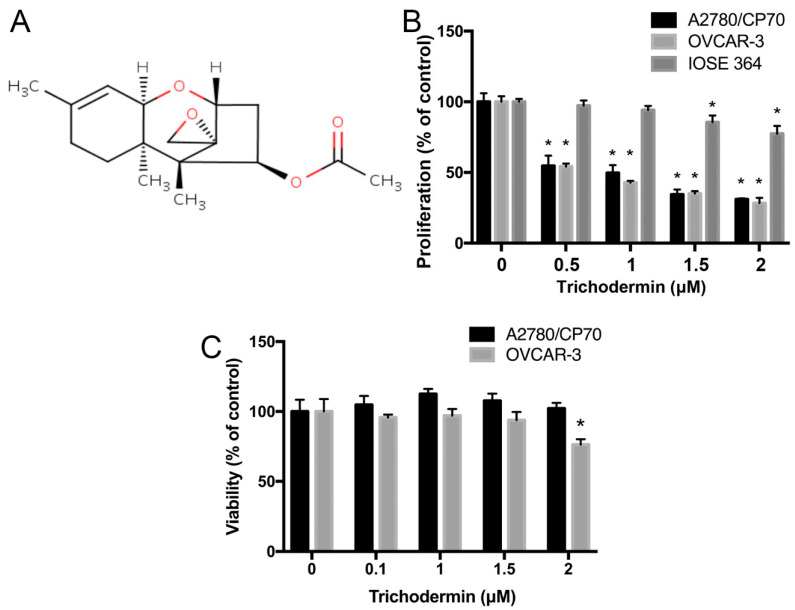
Trichodermin preferentially decreased the proliferation of human ovarian cancer cells. (**A**) Chemical structure of trichodermin. (**B**) The impacts of trichodermin on the proliferation of two human ovarian cancer cell lines (A2780/CP70 and OVCAR-3 cells) and one normal human ovarian surface epithelial cell line (IOSE-364 cells) after 24 h treatment. (**C**) The impacts of trichodermin on the viability of A2780/CP70 and OVCAR-3 cells after 24 h treatment. * *p* < 0.05 compared with the control group.

Fungi are rich sources of bioactive compounds. Many clinical drugs are extracted or derived from fungi. For example, penicillin, one of the most classic antibiotics, was initially obtained from *Penicillium notatum*. Lovastatin, one of the most commonly-used lipid-lowering drugs, was first isolated from *Aspergillus terreus*. So far, some fungal extracts or compounds have been demonstrated to display high anticancer activity [7,8,9]. Chen et al. observed that some *Trichoderma* species exhibited anticancer activity to A549 human lung carcinoma cells and PC3 human prostate cancer cells [10]. Trichodermin (Figure 1A), a trichothecene that specifically inhibits the termination step of protein biosynthesis [11,12], possibly contributed to the observed anticancer activity. Trichodermin showed potential in the induction of apoptosis in MIA PaCa-2 and BxPC-3 human pancreatic cancer cells and JJ012 and SW1353 chondrosarcoma cells [13,14]. Results of toxicology tests indicated that the median lethal doses of trichodermin in mice via oral administration and subcutaneous injection were 1 g/kg and 0.5–1 g/kg, respectively [15]. Compared with doxorubicin [16], a classic chemotherapeutic agent also derived from fungi, the toxicity of trichodermin was much lower. These observations suggested that trichodermin might be a potent anticancer agent with mild side effects. However, whether trichodermin could inhibit ovarian cancer or not was unclear, let alone the underlying mechanisms. In this study, the effects of trichodermin on cell proliferation, apoptosis, cell cycle distribution, and the expression of related proteins in ovarian cancer cells were investigated. To assess the effects of trichodermin on ovarian cancer in vivo, nude mice bearing ovarian cancer xenografts were administrated with trichodermin via intraperitoneal injection and tumor sizes monitored. The results of our study enhance the understanding of the anticancer effects of trichodermin and, perhaps, provide a novel strategy for ovarian cancer treatment.

## 2. Experimental Section

### 2.1. Materials

Trichodermin was kindly provided by Dr. Stephen Cutler from the University of South Carolina. Human ovarian carcinoma cell lines OVCAR-3 and A2780/CP70 were donated by Dr. Bing-Hua Jiang from the Thomas Jefferson University. Human immortalized ovarian surface epithelial cells (IOSE 364) were gifts from Dr. Auersperg from the University of British Columbia. Cells were cultured with RPMI 1640 medium containing 10% fetal bovine serum in a 5% CO_2_ incubator at 37 °C.

Propidium iodide (PI) was obtained from Invitrogen (Carlsbad, CA, USA). Antibodies against cyclin D1, cyclin-dependent kinase 4 (CDK4), CDK2, phosphor-retinoblastoma protein (p-Rb; Ser780), Rb, p16^Ink4a^ (p16), p21^Waf1/Cip1^ (p21), p27^Kip1^ (p27), Cdc25A, phosphor-glycogen synthase kinase-3β (p-GSK-3β; Ser9), and GSK-3β were purchased from Cell Signaling Technology, Inc. (Danvers, MA, USA). Antibodies against c-Myc and glyceraldehyde-3-phosphate dehydrogenase (GAPDH) were purchased from Santa Cruz Biotechnology Inc. (Santa Cruz, CA, USA).

### 2.2. Evaluation of Cell Proliferation and Viability

Cells were seeded into 96-well plates (10^4^/well) and grown overnight. After that, cells were incubated with vehicle or different concentrations of trichodermin for 24 h. Then, the cell proliferation was measured using the CellTiter 96 ^®^ AQueous One Solution Cell Proliferation Assay Kit (Promega Corporation, Madison, WI, USA), and the cell viability was measured using the LDH Cytotoxicity Assay Kit (Beyotime Biotechnology, Nantong, Jiangsu, China). Cell proliferation and viability were presented as a percentage compared to the control group.

### 2.3. Determination of Apoptosis

The annexin V and propidium iodide (PI) staining assay was carried out for the assessment of apoptosis. Briefly, cells were seeded into 6-well plates (10^6^/well), grown overnight, incubated with vehicle or different concentrations of trichodermin for 24 h, washed with phosphate buffer saline (PBS) to remove the residual cell culture medium, stained with annexin V and PI using the Alexa Fluor 488 Annexin V/Dead Cell Apoptosis Kit (Product No. V13241, ThermoFisher Scientific, Waltham, MA, USA) according to the manufacturer’s instructions. Data acquisition and analysis were performed following flow cytometry with accompanying software (FACS Calibur, version 5.1, BD Bioscience, San Jose, CA, USA).

### 2.4. Determination of Cell Cycle Distribution

Cells were treated with vehicle or trichodermin for 24 h, collected, and fixed with cold 70% ethanol at −20 °C overnight. Later, cells were stained with PI, and the cell cycle distribution was analyzed using the flow cytometry (BD Bioscience, San Jose, CA, USA).

### 2.5. Western Blot Assay

Cells were treated with vehicle or trichodermin for 24 h, and then cell proteins were extracted. The western blot assay was conducted based on a published method [17].

### 2.6. Plasmid Transfection

Plasmid transfection was conducted with the jetPRIMETM DNA and siRNA transfection reagent (VWR International, Radnor, PA, USA). Cells were transfected with either pMXs-hc-MyC (Addgene plasmid #17220) or pMXS-Puro (Addgene plasmid #69474, as the empty control plasmid). 4 h later, cells were exposed to vehicle or 1 μM trichodermin for 24 h for further tests.

### 2.7. Animal Experiments

All experiments with mice were approved by the Marshall University Institutional Animal Care and Use Committee (Marshall University IACUC 705). Four-to-six-week-old female nude mice (BALB/c nude; strain code 194 (homozygous) Charles River Laboratories; N = 14) were used. The mice were housed in autoclaved cages with food and water in HEPA-filtered racks. The mice were acclimatized for one week.

OVCAR-3 human ovarian cancer cells (5 × 10^6^) were resuspended in a 0.2 mL solution of 1:1 Matrigel (*v*/*v*) and serum-free RPMI 1640 medium and implanted subcutaneously between the scapula of nude mice. Mice were monitored at least weekly until a palpable tumor nodule was present. Once palpable tumors formed and reached a minimum measurable volume (30–60 mm^3^), mice were randomized and divided into two groups (i.e., the vehicle group and the trichodermin group), and the four-week vehicle/trichodermin treatment was started.

Mice were intraperitoneally injected three times weekly with 0.2 mL vehicle (sterile PBS) or trichodermin (5 mg/kg body weight, dissolved in pre-warmed sterile PBS). The tumor size was measured three times weekly using calipers, and the tumor volume was calculated by the formula: length (L) × width (w)^2^ × (1/2). Mouse weight, food, and water intake were measured weekly.

### 2.8. Statistical Analysis

All data are expressed as mean ± standard error of mean (SEM) for three independent experiments. One-way analysis of variance (ANOVA) followed by a post-hoc test was used for statistical significance analysis (SPSS software, Version 22.0, IBM, Armonk, NY, USA). The difference in the tumor size between the two groups was analyzed with the Mann–Whitney U test. *p* values less than 0.05 were considered statistically significant.

## 3. Results

### 3.1. Trichodermin Specifically Lowers the Proliferation of Ovarian Cancer Cells

Trichodermin dose-dependently decreased the proliferation of OVCAR-3 and A2780/CP70 ovarian cancer cells (Figure 1B). The half-maximal inhibitory concentrations (IC_50_) of trichodermin in OVCAR-3 and A2780/CP70 ovarian cancer cell lines were 0.73 μM and 0.65 μM, respectively. Since OVCAR-3 cells were cisplatin-sensitive while A2780/CP70 were cisplatin-resistant, our results indicated that trichodermin had the potential in overcoming cisplatin-resistant. The anti-proliferation effect of trichodermin on normal ovarian surface epithelial cells (IOSE-364) was much less than on the cancer cell lines (Figure 1B). When exposed to trichodermin at the final concentration of 2 μM for 24 h, the proliferation of IOSE-364 cells was about 80%. The estimated IC_50_ of trichodermin in IOSE-364 cells was 3.67 μM.

Although trichodermin effectively inhibited the proliferation of both ovarian cancer cells, it weakly affected the viability of both ovarian cancer cells (Figure 1C).

Taken together, trichodermin mainly inhibited A2780/CP70 and OVCAR-3 ovarian cancer cells via suppressing the proliferation rather than being cytotoxic.

### 3.2. Trichodermin Suppresses Ovarian Cancer Cells Mainly via Inducing G0/G1 Cell Cycle Arrest

Inducing apoptosis is an important strategy for cancer treatment. Former studies suggested that trichodermin activated apoptosis in some types of cancer cells (e.g., BxPC-3 cells and SW1353 cells) [13,14]. However, trichodermin showed weak effects on apoptosis in ovarian cancer cells (Figure S1). The percentages of annexin V(+) only (indicating early apoptosis) cells and annexin V(+) plus PI (+) (indicating late apoptosis) cells were not significantly increased after the trichodermin treatment. These results implied that inducing apoptosis was not the predominant anticancer mechanism of trichodermin in ovarian cancer cells.

**Figure 2:**
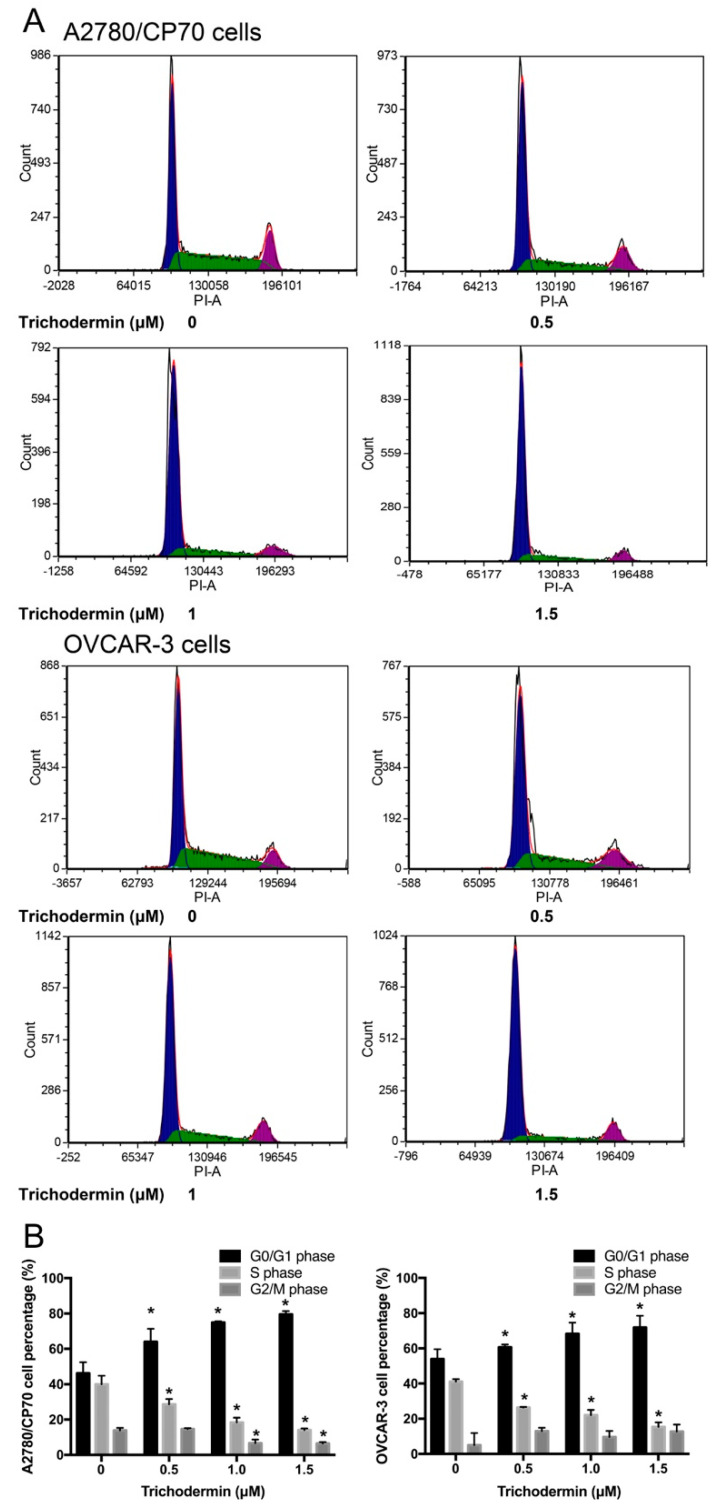
Trichodermin induced G0/G1 cell cycle arrest in A2780/CP70 and OVCAR-3 cells after 24 h treatment. The cell distribution was measured using flow cytometry. (**A**) Flow cytometry plots. (**B**) The cell cycle distribution of A2780/CP70 cells and OVCAR-3 cells after trichodermin treatment. * *p* < 0.05 compared with the control group.

Interrupting the cell cycle process is another key strategy for cancer treatment. In this study, trichodermin dramatically increased the proportion of cells in the G0/G1 phase and decreased the proportion of cells in the S phase (Figure 2). In OVCAR-3 cells, the proportion of cells in the G0/G1 phase increased from 53% to 72% after exposure to 1.5 μM trichodermin for 24 h, while the proportion of cells in the S phase decreased from 41% to 15%. In A2780/CP70 cells, similar effects were detected. These results indicated that trichodermin significantly induced G0/G1 cell cycle arrest in ovarian cancer cells.

### 3.3. Trichodermin Inhibits Cyclin-Dependent Kinases (CDKs) and Cyclins

**Figure 3:**
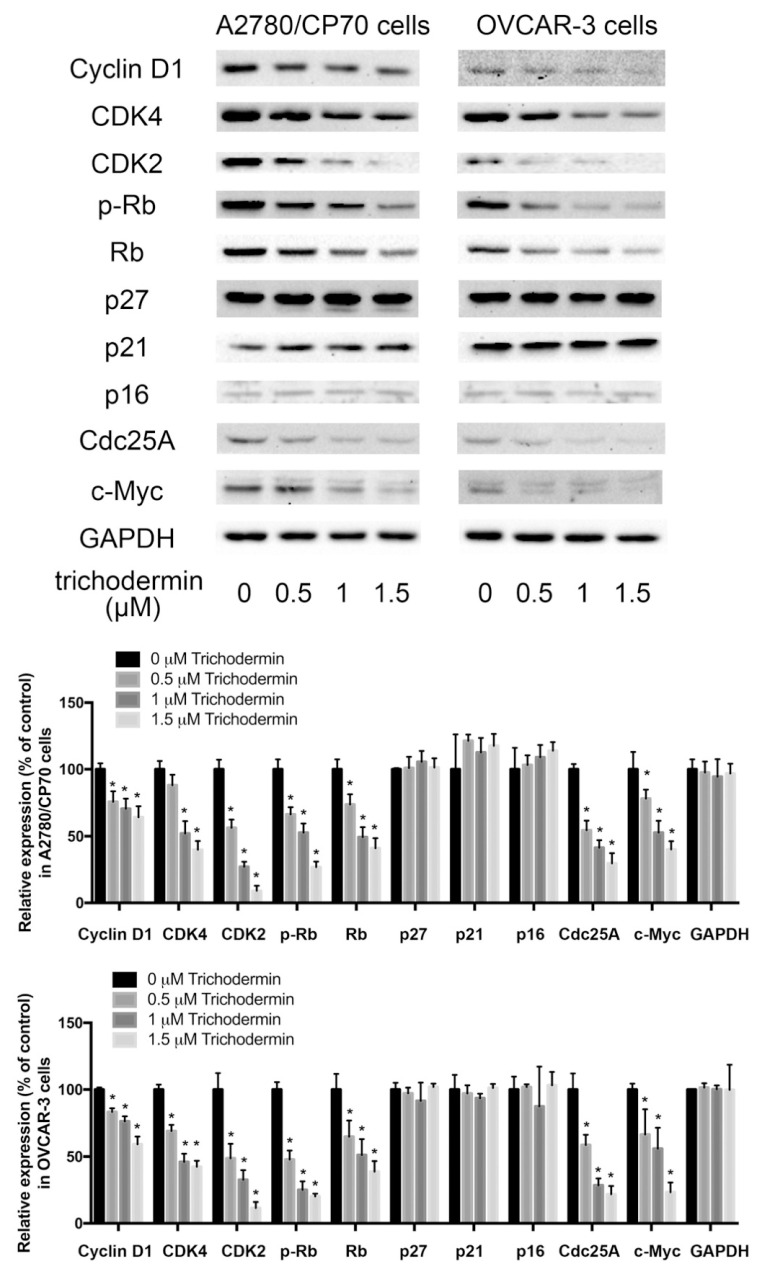
Trichodermin suppressed the expression of cyclin D1, cyclin-dependent kinases (CDKs), phosphor-retinoblastoma protein (p-Rb), Rb, Cdc25A, and c-Myc after 24 h treatment. However, it minimally affected the expression of CDK inhibitors (CDKIs), including p27^Kip1^ (p27), p21^Waf1/Cip1^ (p21), and p16^Ink4a^ (p16). Glyceraldehyde-3-phosphate dehydrogenase (GAPDH) served as the loading control. * *p* < 0.05 compared with the control group.

CDKs and cyclins cooperate to regulate cell-cycle progression. Cyclin D-CDK4/6 and cyclin E-CDK2 complexes are important for the G1/S transition. Once inhibited, the cell cycle halts in the G1 phase. Both complexes can phosphorylate Rb, resulting in the release of transcription factor E2F from the Rb-E2F complex and the enhanced expression of downstream target genes. The results of the western blot assay indicated that trichodermin significantly suppressed the expression of cyclin D1, CDK4, CDK2, p-Rb, and Rb (Figure 3) in both ovarian cancer cell lines.

The activity of CDKs can be regulated by small polypeptide inhibitors called CDK inhibitors (CDKIs). CDKIs directly bind to and inactivate CDKs. Ink4 and Cip/Kip are two families of CDKIs. Members of the Ink4 family, including p16^Ink4a^, p15^Ink4b^, p18^Ink4c^, and p19^Ink4d^, bind exclusively to CDK4 and CDK6. Members of the Cip/Kip family, including p21^Waf1/Cip1^, p27^Kip1^, and p57^Kip2^, show little selectivity in binding to and inhibition of CDKs. In this study, no significant changes were observed in the expression of p16, p21, and p27 in both ovarian cancer cell lines, implying trichodermin did not regulate the activity of CDKs by increasing these CDKIs.

Besides CDKIs, the activity of CDKs can be affected by phosphatases through modification of their phosphorylation status. Cdc25A is a dual-specificity protein phosphatase that mediates the removal of N-terminal inhibitory phosphates of the G1 CDKs, including CDK4, CDK6, and CDK2 [18]. It plays the rate-limiting role for the S phase entry [18]. Overexpression of Cdc25A accelerates the G1/S transition [19]. Trichodermin down-regulated the expression of Cdc25A in both ovarian cancer cell lines, which hampered the G1/S transition and resulted in the G1 cell cycle arrest.

The above findings suggested that trichodermin inhibited the expression of G1 CDKs and cyclins, and it inhibited CDKs via suppressing Cdc25A rather than promoting CDKIs.

### 3.4. Trichodermin Induces G0/G1 Cell Cycle Arrest via Targeting c-Myc

**Figure 4:**
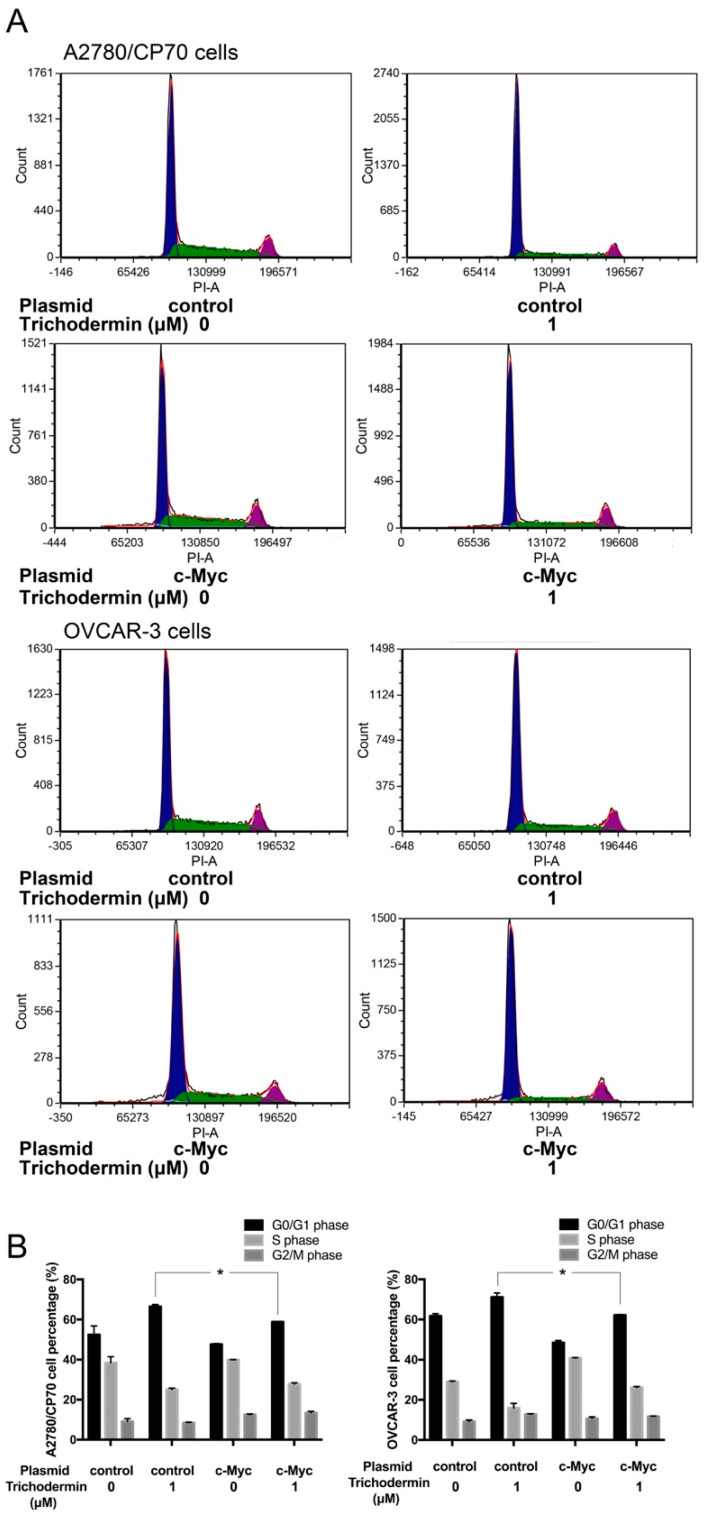
Overexpression of c-Myc attenuated trichodemin-induced G0/G1 cell cycle arrest in A2780/CP70 and OVCAR-3 cells. (**A**) Flow cytometry plots. (**B**) The cell cycle distribution of A2780/CP70 cells and OVCAR-3 cells after plasmid transfection and trichodermin treatment. * *p* < 0.05 compared with the specific group.

**Figure 5:**
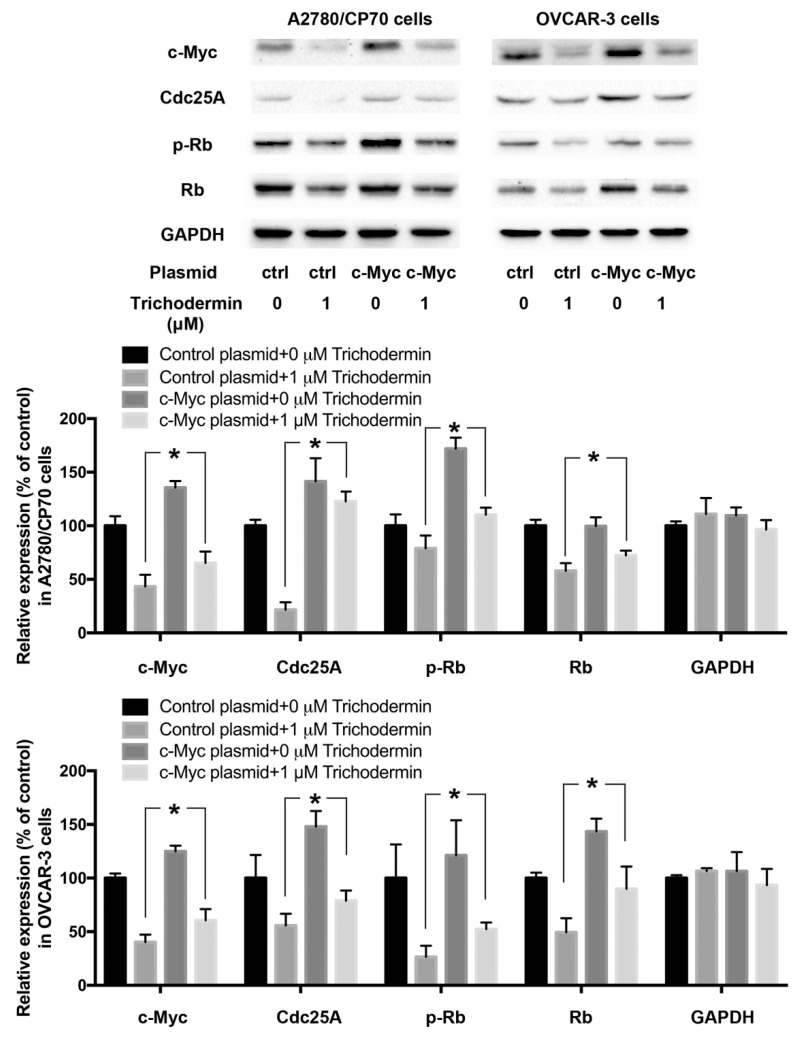
Overexpression of c-Myc attenuated trichodermin-induced suppression of c-Myc, Cdc25A, phosphor-retinoblastoma protein (p-Rb), and Rb in A2780/CP70 and OVCAR-3 cells. Glyceraldehyde-3-phosphate dehydrogenase (GAPDH) served as the loading control. * *p* < 0.05 compared with the specific group.

c-Myc is a transcription factor that regulates the growth and cell cycle by inducing the expression of genes required for these processes. Overexpression of c-Myc increases the rate of tumor formation. A small increase in the c-Myc level can affect cell cycle progression [19]. Almost 50% of human cancers show dysregulation or activation of c-Myc [20]. In addition, overexpression of c-Myc was found in 63.5% of serous ovarian tumors [21]. c-Myc stimulates proliferation partially by promoting entry into the S phase. It coordinates the signaling that governs the G1/S transition and enhances the metabolic activity needed to execute genome replication [22]. Several cell cycle-related proteins, such as CDK4, Cdc25A, and E2F2, are known to be downstream targets of c-Myc [22]. Trichodermin remarkably inhibited the expression of c-Myc. Plasmid transfection experiments revealed that overexpression of c-Myc in A2780/CP70 and OVCAR-3 cells attenuated the effect of trichodermin on inducing G0/G1 cell cycle arrest (Figure 4), and it rescued the trichodermin-mediated decreased expression of Cdc25A, p-Rb, and Rb (Figure 5). These findings demonstrated that c-Myc was partially involved in the induction of G0/G1 cell cycle arrest by trichodermin in ovarian cancer cells.

### 3.5. Trichodermin Reduces Ovarian Tumor Growth in Xenograft-Bearing Mice

**Figure 6:**
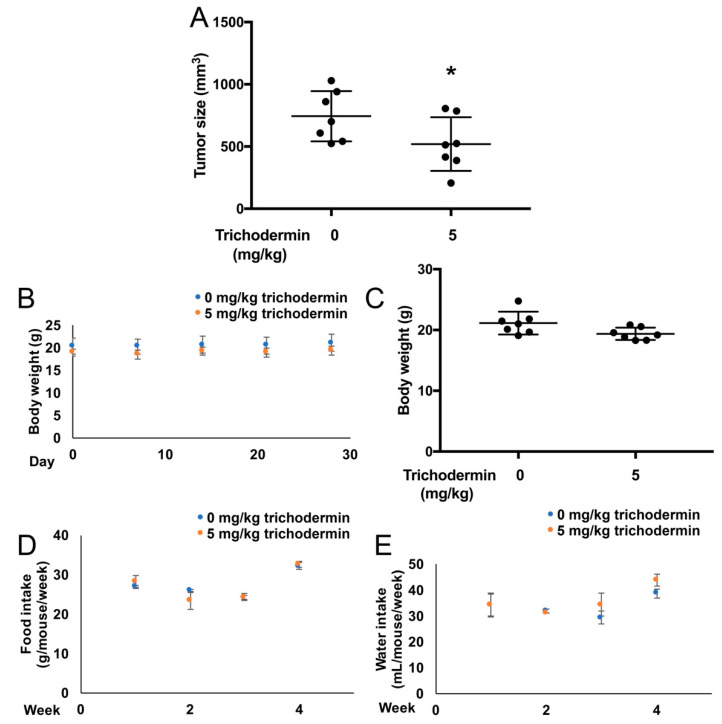
Trichodermin inhibited the growth of OVCAR-3 xenografts in BALB/C nude mice. OVCAR-3 human ovarian cancer cell suspension (containing 5 × 10^6^ cells) was mixed with Matrigel (1:1, *v*/*v*) and implanted subcutaneously between the scapula of nude mice. After palpable tumors were formed (tumor size > 30–60 mm^3^), mice were injected three times weekly with 0.2 mL vehicle (sterile PBS) or trichodermin (5 mg/kg body weight, dissolved in pre-warmed sterile PBS) for four weeks. (**A**) Tumor size at Day 28 of trichodermin (5 mg/kg body weight) or vehicle treatment. (**B**) Body weight during the four-week treatment of trichodermin (5 mg/kg body weight) or vehicle. (**C**) Body weight at Day 28 of trichodermin (5 mg/kg body weight) or vehicle treatment. (**D**) Food intake and (**E**) water intake during the four-week treatment of trichodermin (5 mg/kg body weight) or vehicle. * *p* < 0.05 compared with the control group.

To investigate the role of trichodermin in vivo, a subcutaneous mice model was used. According to the reference, OVCAR-3 cells are highly metastatic in the intraperitoneal mice model [23]. This study focused on the impact of trichodermin on the growth of ovarian cancers and was not involved in assessing the anti-metastatic activity of trichodermin, the subcutaneous model but not the intraperitoneal model was chosen to avoid the complexity of metastasis. Briefly, OVCAR-3 cells were mixed with Matrigel and implanted subcutaneously in BALB/c nude mice. After palpable tumors were formed and reached a minimum measurable volume (30–60 mm^3^), the xenograft-bearing mice were given a four-week treatment of vehicle or trichodermin. The tumor size was measured three times per week. No mice died during the experiment. The results (Figure 6) revealed that the average tumor size in the trichodermin-treated group was significantly smaller than that in the vehicle-treated group. The body weights, food intake, and water intake were not significantly different between the two groups. Although the subcutaneous model is not the most clinically relevant model, these results gained preliminary insight into the in vivo therapeutic potential of trichodermin to inhibit ovarian cancer growth. In the future, the anti-ovarian cancer activities of trichodermin in the intraperitoneal mice model, which is a more clinically relevant model, will be investigated to further evaluate the potential of trichodermin in ovarian cancer treatment.

## 4. Discussion

Despite improvements in diagnosis and medical treatment, ovarian cancer is still a deadly disease for women. According to the GOG-0218 double-blind, placebo-controlled phase III trial, the median progression-free survival (PFS) of previously untreated, incompletely resectable stage III or any stage IV ovarian cancer patients with traditional chemotherapeutic drugs was 10.3 months [24]. The use of traditional chemotherapeutic drugs plus bevacizumab, a targeted drug that neutralized the vascular endothelial growth factor, largely prolongs the median PFS of ovarian cancer patients [24]. However, the prolonged PFS was 14.1 months [24]. Therefore, it is important and necessary to find novel strategies to treat ovarian cancer.

Trichodermin is a small molecule first isolated from *Trichoderma viride* strain in the mid-60s [25]. It displays inhibitory activity against many plant fungal pathogens (e.g., *Botrytis cinerea* and *Magnaporthe oryzae*) [26] and yeast [12]. As a protein synthesis inhibitor, trichodermin mainly disturbs elongation and termination processes [12,27]. It has a high affinity to the donor and the acceptor site of the peptidyl transferase center of eukaryotic ribosomes [28,29]. Due to this property, it is possible that trichodermin might have anti-cancer activity. Previous studies revealed that trichodermin suppresses the proliferation of HCT15 colon cancer cells [30], MIA PaCa-2 and BxPC-3 pancreatic cancer cells [13], and JJ012 and SW1353 chondrosarcoma cells [14]. It triggers apoptosis via the mitochondrial pathway and the endoplasmic reticulum stress-mediated pathway in JJ012 and SW1353 chondrosarcoma cells [14]. Trichodermin triggers DNA damage stress to activate p53 for executing apoptosis in p53-mutated MIA PaCa-2 and BxPC-3 pancreatic cancer cells [13]. Additionally, it increases apoptosis through mitotic arrest in MIA PaCa-2 and BxPC-3 pancreatic cancer cells, and C-Jun N-terminal kinase (JNK) is identified as one of the targets of trichodermin [13].

Different from former findings, the induction of the G0/G1 cell cycle arrest rather than apoptosis contributes to the inhibitory effects of trichodermin in the ovarian cancer cell lines used in this study. Cancer is a disease of cell proliferation, whereby cancer cells progressively and inexorably lose normal cell cycle control [31]. The length of a cell cycle determines the rate of proliferation. The cell cycle of a non-quiescent eukaryotic cell is divided into four phases, which are the G1 phase, S phase, G2 phase, and M phase. The length of the cell cycle varies in different cell types and heavily relies on the length of time spent within the G1 phase [32]. The transition of each phase inside the cell cycle is controlled by cell cycle checkpoints. There are three main cell cycle checkpoints, including the G1/S checkpoint, the intra-S checkpoint, and the G2/M checkpoint. At each checkpoint, the cell determines whether it is ready for progression to the next phase and halts progress if conditions are unfavorable [33]. CDKs are a family of protein serine/threonine kinases that drive each of the major cell cycle transition points (G1, S, G2, M), inducing downstream processes by phosphorylating specific proteins. Their activity depends on the association with cyclins and CDKIs. CDK activity is activated by binding to cyclins. CDKIs negatively regulate the activity of CDKs by directly binding to CDKs. The Cdc25 phosphatases also regulate the activity of CDKs. They remove the inhibitory phosphates from Thr-14 and Tyr-15 in CDKs, which activates the kinases and drives the progression through the cell cycle. Both Cdc25A and Cdc25B are potential human oncogenes and are overexpressed in a number of tumors [34]. Each phase is controlled by different CDK/cyclin pairs. Early in the G1 phase, growth factors stimulate the synthesis of G1 cyclins, represented by the cyclin D family of cyclins. The G1 cyclins activate CDK4/6 to induce the synthesis of downstream targets, one of which is cyclin E. The rise in the level of cyclin E and the activity of its partner, CDK2, drive the cell past the G1/S checkpoint, after which the cell is irreversibly committed to proceeding to DNA synthesis (the M phase). In this study, trichodermin decreased the levels of two G1 CDKs (i.e., CDK2 and CDK4), which was consistent with its function on inducing the G0/G1 cell cycle arrest. Trichodermin’s inhibitory effects on CDKs were associated with the reduction of cyclin D1 and Cdc25A. The expression of CDKIs was not influenced by trichodermin.

c-Myc was identified as an upstream protein of Cdc25A. Trichodermin effectively inhibited the expression of c-Myc. Overexpression of c-Myc attenuated the effects of trichodermin on inducing G0/G1 cell cycle arrest. It implied that c-Myc is a vital target for trichodermin. The c-Myc gene is amplified in 30% to 60% of human ovarian tumors [35]. A higher level of c-Myc is related to the decreased disease-free survival and overall survival in ovarian cancer patients [35]. c-Myc specific siRNA significantly reduces the growth of ovarian tumors [35]. As a transcription factor, c-Myc requires the basic helix-loop-helix partner protein, Max, to bind specific DNA sequences to solidify its role in transcription [36]. c-Myc regulates transcription through several mechanisms, including recruitment of histone acetylases, chromatin modulating proteins, basal transcriptional factors, and DNA methyltransferase. It is reported that up to about 15% of genes in genomes are regulated by c-Myc [36]. However, only parts of the genes are universally regulated by c-Myc independent of cell type or species. c-Myc consistently represses genes involved in growth arrest and apoptosis. Meanwhile, it directly promotes genes involved in cell cycle progression. Cdc25, cyclin D1, and CDK4 are identified as consistently emerged targets of c-Myc. Hence, it accelerates entry into the S-phase of the cell cycle. Our findings are supportive of this. In addition to genes directly related to the cell cycle, c-Myc also increases protein synthesis. A number of ribosomal protein genes have been identified as direct c-Myc targets. Cell proliferation requires the cooperation of multiple proteins. Overexpression of c-Myc accelerates protein synthesis, producing large amounts of proteins for the rapid proliferation of cells. Previously, trichodermin has been shown to bind to ribosomes to interrupt the elongation and termination of protein synthesis. A decrease in the expression of c-Myc might aggravate the impairment of protein synthesis, which exacerbates the contradiction between the demand of protein required for cell proliferation and the actual ability of cells in protein synthesis, resulting in cell cycle arrest and a lowered proliferation rate. Further experiments are needed to prove this hypothesis.

As c-Myc is such an important transcription factor for cells, it is exquisitely regulated. Many upstream proteins of c-Myc are found overexpressed in ovarian tumors, one of which is glycogen synthase kinase 3β (GSK-3β) [37]. GSK-3β is a serine/threonine-protein kinase involved in a wide variety of processes. Active GSK-3β phosphorylates β-catenin and, thereby, triggers its proteasomal degradation [38], which keeps relatively low levels of β-catenin in cells under normal conditions. When phosphorylated at the N-terminal Serine 9 (Ser9) residue, GSK-3β is inactivated [39], leading to the accumulation of β-catenin. β-catenin is a critical component of the Wnt signal transduction pathway. As a transcription factor, it regulates the expression of various genes, including c-Myc and cyclin D1. Aberrant expression of β-catenin induces the malignant transformation of normal cells, and its abnormal activity has been reported in many cancer types [40]. Both p-GSK-3β and β-catenin were found to be upregulated in ovarian tumors, implying they perform a positive role in the progression of ovarian cancer. Trichodermin effectively lowered the expression of p-GSK-3β but not GSK-3β, indicating that it restored the activity of GSK-3β in ovarian cancer cells (Figure S2). Zhang et al. found that secalonic acid D, which was a mycotoxin, induced apoptosis, and G1 cell cycle arrest via the GSK-3 β/β-catenin/c-Myc pathway in leukemia cells [41]. Our results suggested that trichodermin might also inhibit ovarian cancer cells by targeting this pathway. Additional experiments are required to further confirm this hypothesis.

## 5. Conclusions

In conclusion, our research confirmed the effectiveness of trichodermin against ovarian cancer in vitro and in vivo. It predominantly inhibited ovarian cancer cells by inducing G0/G1 cell cycle arrest via suppressing the c-Myc/Cdc25A pathway. These results provided evidence of the anti-ovarian cancer activity of trichodermin and suggested that trichodermin might be applied as adjuvant treatment for ovarian cancer in the future. More studies are required to determine its effectiveness and safety in animals and humans and to further understand any additional mechanism(s) of action.

## Data Availability

All the data used in this study have been provided in the main text.

## Supplementary Materials

The following are available online at https://www.mdpi.com/article/10.3390/ijms22095022/s1.

## References

[1] Siegel R.L., Miller K.D., Fuchs H.D., Jemal A. (2021). Cancer statistics, 2021. CA Cancer J. Clin..

[2] Morgan R.J., Alvarez R.D., Armstrong D.K., Burger R.A., Chen L.M., Copeland L., Crispens M.A., Gershenson D.M., Gray H.J., Hakam A. (2013). Ovarian cancer, version 2. 2013. J. Natl. Compr. Cancer Netw..

[3] Targeted Cancer Therapies. https://www.cancer.gov/about-cancer/treatment/types/targeted-therapies/targeted-therapies-fact-sheet#what-are-the-side-effects-of-targeted-cancer-therapies.

[4] McDonald M.E., Salinas E.A., Devor E.J., Newtson A.M., Thiel K.W., Goodheart M.J., Bender D.P., Smith B.J., Leslie K.K., Gonzalez-Bosquet J. (2019). Molecular characterization of non-responders to chemotherapy in serous ovarian cancer. Int. J. Mol. Sci..

[5] Chase D.M., Mathur N., Tewari K.S. (2010). Drug discovery in ovarian cancer. Recent Pat. Anticancer Drug Discov..

[6] Cai J., Ma H., Huang F., Zhu D., Bi J., Ke Y., Zhang T. (2013). Correlation of bevacizumab-induced hypertension and outcomes of metastatic colorectal cancer patients treated with bevacizumab: A systematic review and meta-analysis. World J. Surg. Oncol..

[7] Cheng S., Swanson K., Eliaz I., McClintick J.N., Sandusky G.E., Sliva D. (2015). Pachymic acid inhibits growth and induces apoptosis of pancreatic cancer in vitro and in vivo by targeting er stress. PLoS ONE.

[8] Deng Q., Yu X., Xiao L., Hu Z., Luo X., Tao Y., Yang L., Liu X., Chen H., Ding Z. (2013). Neoalbaconol induces energy depletion and multiple cell death in cancer cells by targeting pdk1-pi3-k/akt signaling pathway. Cell Death Dis..

[9] Li B., Gao Y., Rankin G.O., Rojanasakul Y., Cutler S.J., Tu Y., Chen Y.C. (2015). Chaetoglobosin k induces apoptosis and g2 cell cycle arrest through p53-dependent pathway in cisplatin-resistant ovarian cancer cells. Cancer Lett..

[10] Chen X., Lei B.X., Wen T.C., Zeng Q. (2017). The anticancer activity of endophytic fungi *trichoderma* sp. from *nothapodytes pittosporoides*. Lishizhen Med. Mater. Med. Res..

[11] Malmierca M.G., Cardoza R.E., Alexander N.J., McCormick S.P., Hermosa R., Monte E., Gutierrez S. (2012). Involvement of trichoderma trichothecenes in the biocontrol activity and induction of plant defense-related genes. Appl. Environ. Microbiol..

[12] Stafford M.E., McLaughlin C.S. (1973). Trichodermin, a possible inhibitor of the termination process of protein synthesis. J. Cell Physiol..

[13] Su C.M., Wang S.W., Lee T.H., Tzeng W.P., Hsiao C.J., Liu S.C., Tang C.H. (2013). Trichodermin induces cell apoptosis through mitochondrial dysfunction and endoplasmic reticulum stress in human chondrosarcoma cells. Toxicol. Appl. Pharmacol..

[14] Chien M.H., Lee T.H., Lee W.J., Yeh Y.H., Li T.K., Wang P.C., Chen J.J., Chow J.M., Lin Y.W., Hsiao M. (2017). Trichodermin induces c-jun n-terminal kinase-dependent apoptosis caused by mitotic arrest and DNA damage in human p53-mutated pancreatic cancer cells and xenografts. Cancer Lett..

[15] Culter H.G., LeFiles J.H. (1978). Trichodermin: Effects on plants. Plant Cell Physiol..

[16] Gol’dberg L.E., Filippos’iants S.T., Shepelevtseva N.G., Vertogradova T.P. (1983). Toxicity, pharmacokinetics and pharmacodynamics of soviet-made doxorubicin. Antibiotiki.

[17] Gao Y., Rankin G.O., Tu Y., Chen Y.C. (2016). Theaflavin-3, 3′-digallate decreases human ovarian carcinoma ovcar-3 cell-induced angiogenesis via akt and notch-1 pathways, not via mapk pathways. Int. J. Oncol..

[18] Sexl V., Diehl J.A., Sherr C.J., Ashmun R., Beach D., Roussel M.F. (1999). A rate limiting function of cdc25a for s phase entry inversely correlates with tyrosine dephosphorylation of cdk2. Oncogene.

[19] Blomberg I., Hoffmann I. (1999). Ectopic expression of cdc25a accelerates the g(1)/s transition and leads to premature activation of cyclin e- and cyclin a-dependent kinases. Mol. Cell Biol..

[20] Salvi N. (2019). Intrinsically Disordered Proteins Dynamics, Binding, and Function.

[21] Tashiro H., Miyazaki K., Okamura H., Iwai A., Fukumoto M. (1992). C-myc over-expression in human primary ovarian tumours: Its relevance to tumour progression. Int. J. Cancer.

[22] Mendelsohn J. (2008). The Molecular Basis of Cancer.

[23] Mitra A.K., Davis D.A., Tomar S., Roy L., Gurler H., Xie J., Lantvit D.D., Cardenas H., Fang F., Liu Y.Y. (2015). In vivo tumor growth of high-grade serous ovarian cancer cell lines. Gynecol. Oncol..

[24] Burger R.A., Brady M.F., Bookman M.A., Fleming G.F., Monk B.J., Huang H., Mannel R.S., Homesley H.D., Fowler J., Greer B.E. (2011). Incorporation of bevacizumab in the primary treatment of ovarian cancer. N. Engl. J. Med..

[25] Godtfredsen W.O., Vangedal S. (1965). Trichodermin, a new sesquiterpene antibiotic. Acta Chem. Scand..

[26] Wang G.P., Zheng B.Q., Zhou Z.Z., Zhang C.L. (2010). Optimization of fermentation conditions for trichodermin by the mutant strain ul60-11 of *trichoderma taxi*. Chin. J. Biol. Control.

[27] Wei C.M., Campbell I.M., McLaughlin C.S., Vaughan M.H. (1974). Letter: Binding of trichodermin to mammalian ribosomes and its inhibition by other 12,13-epoxytrichothecenes. Mol. Cell Biochem..

[28] Barbacid M., Vazquez D. (1974). Binding of (acetyl-14c)trichodermin to the peptidyl transferase centre of eukaryotic ribosomes. Eur. J. Biochem..

[29] Carrasco L., Barbacid M., Vazquez D. (1973). The trichodermin group of antibiotics, inhibitors of peptide bond formation by eukaryotic ribosomes. Biochim. Biophys. Acta.

[30] Choi S.U., Choi E.J., Kim K.H., Kim N.Y., Kwon B.M., Kim S.U., Bok S.H., Lee S.Y., Lee C.O. (1996). Cytotoxicity of trichothecenes to human solid tumor cells in vitro. Arch. Pharm. Res..

[31] Quaranta V., Tyson D., Frick P., Dubitzky W., Wolkenhauer O., Cho K.-H., Yokota H. (2013). Cell cycle, cancer cell cycle and oncogene addiction. Encyclopedia of Systems Biology.

[32] Shehata M., Waterhouse P.D., Casey A.E., Fang H., Hazelwood L., Khokha R. (2018). Proliferative heterogeneity of murine epithelial cells in the adult mammary gland. Commun. Biol..

[33] Nghiem P., Park P.K., Kim Y.S., Vaziri C., Schreiber S.L. (2001). ATR inhibition selectively sensitizes G1 checkpoint-deficient cells to lethal premature chromatin condensation. Proc. Natl. Acad. Sci. USA.

[34] Zhang Z.Y. (2003). Mechanistic studies on protein tyrosine phosphatases. Prog. Nucleic Acid Res. Mol. Biol..

[35] Reyes-Gonzalez J.M., Armaiz-Pena G.N., Mangala L.S., Valiyeva F., Ivan C., Pradeep S., Echevarria-Vargas I.M., Rivera-Reyes A., Sood A.K., Vivas-Mejia P.E. (2015). Targeting c-myc in platinum-resistant ovarian cancer. Mol. Cancer Ther..

[36] Dang C.V., O’Donnell K.A., Zeller K.I., Nguyen T., Osthus R.C., Li F. (2006). The c-myc target gene network. Semin. Cancer Biol..

[37] Novetsky A.P., Thompson D.M., Zighelboim I., Thaker P.H., Powell M.A., Mutch D.G., Goodfellow P.J. (2013). Lithium chloride and inhibition of glycogen synthase kinase 3beta as a potential therapy for serous ovarian cancer. Int. J. Gynecol. Cancer.

[38] Huang L., Chen D., Liu D., Yin L., Kharbanda S., Kufe D. (2005). Muc1 oncoprotein blocks glycogen synthase kinase 3beta-mediated phosphorylation and degradation of beta-catenin. Cancer Res..

[39] Xu X., Zou L., Yao Q., Zhang Y., Gan L., Tang L. (2017). Silencing dek downregulates cervical cancer tumorigenesis and metastasis via the dek/p-ser9-gsk-3beta/p-tyr216-gsk-3beta/beta-catenin axis. Oncol. Rep..

[40] Averett C., Arora S., Zubair H., Singh S., Bhardwaj A., Singh A.P. (2014). Chapter nine—Molecular targets of honokiol: A promising phytochemical for effective cancer management. Enzymes.

[41] Zhang J.Y., Tao L.Y., Liang Y.J., Yan Y.Y., Dai C.L., Xia X.K., She Z.G., Lin Y.C., Fu L.W. (2009). Secalonic acid d induced leukemia cell apoptosis and cell cycle arrest of g(1) with involvement of gsk-3beta/beta-catenin/c-myc pathway. Cell Cycle.

