# Supplementary material for: Trichodermin Induces G0/G1 Cell Cycle Arrest by Inhibiting c-Myc in Ovarian Cancer Cells and Tumor Xenograft-Bearing Mice
Source: Int J Mol Sci. 2021 May 9;22(9):5022. doi: 10.3390/ijms22095022 (PMC8126000; doi:10.3390/ijms22095022)
Supplement: Supplementary file 1 [file ijms-22-05022-s001.zip › ijms-1188800-supplementary.pdf]

Supplementary materials

Figure S1

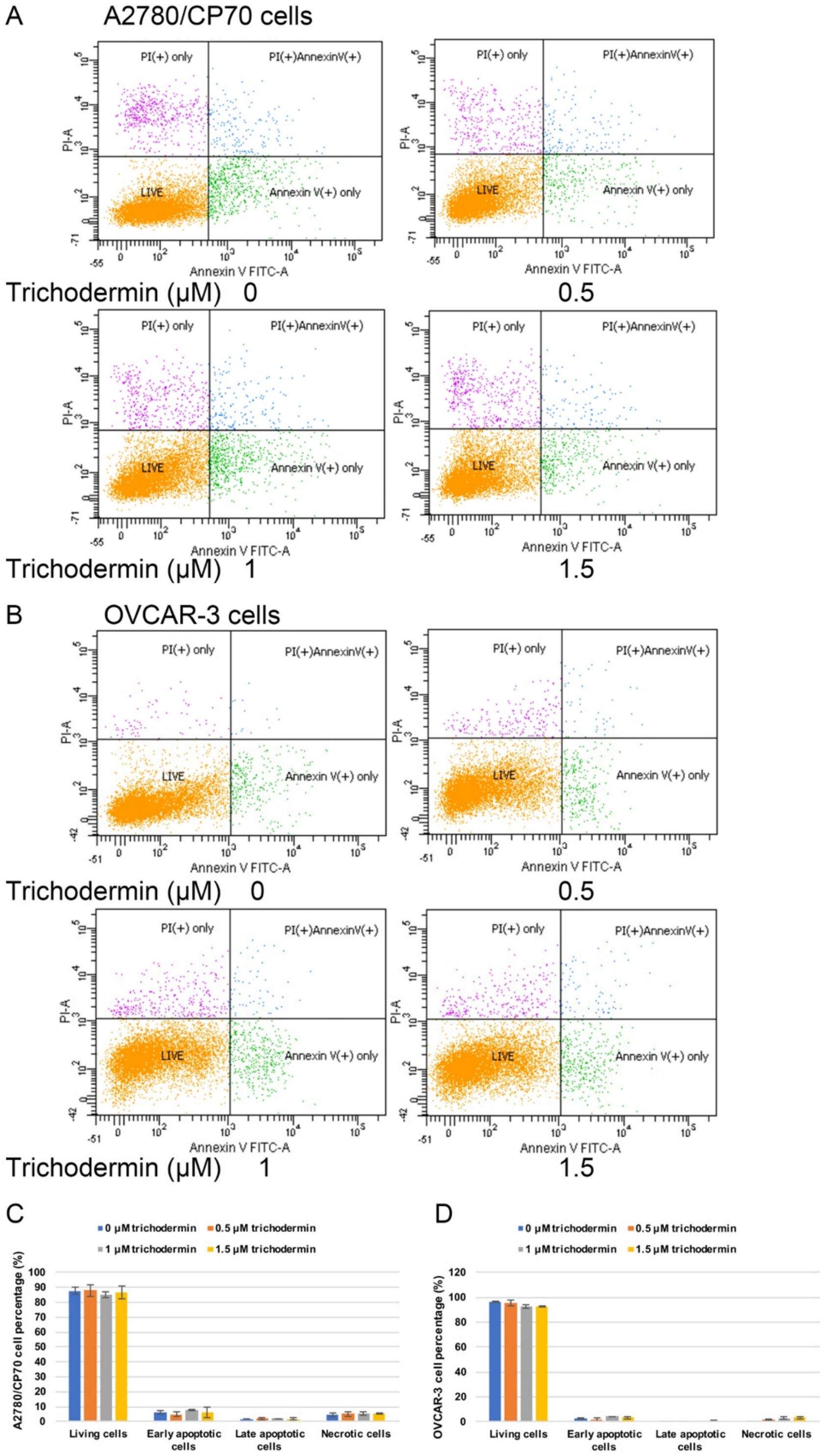

Figure S1. Trichodermin minimally induced apoptosis in A2780/CP70 and OVCAR-3 cells after 24h treatment (Annexin V and PI staining assay by flow cytometry).

Figure S2

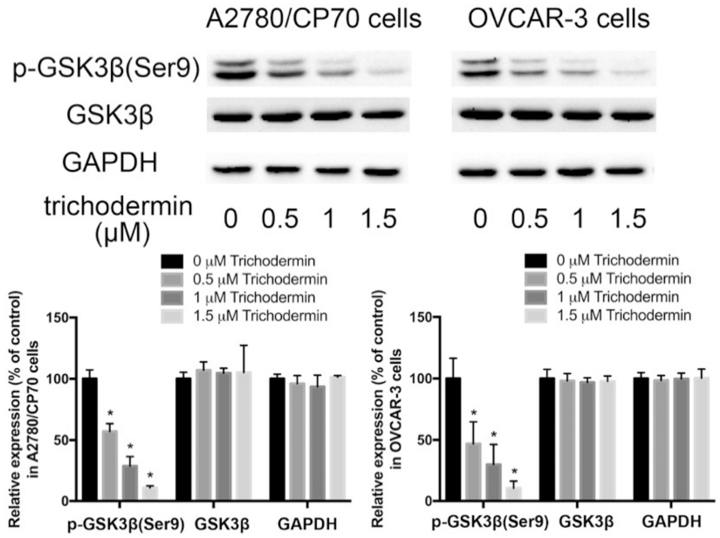

Figure S2. Trichodermin decreased the phosphorylation of glycogen synthase kinase 3β (GSK-3β) at Ser9 in A2780/CP70 and OVCAR-3 cells after 24h treatment.

Simple Summary: It is vitally important that scientists are able to describe their work simply and concisely to the public, especially in an open-access on-line journal. The simple summary consists of no more than 200 words in one paragraph and contains a clear statement of the problem addressed, the aims and objectives, pertinent results, conclusions from the study and how they will be valuable to society. This should be written for a lay audience, i.e., no technical terms without explanations. No references are cited and no abbreviations. Submissions without a simple summary will be returned directly. Example could be found at http://www.mdpi.com/2076-2615/6/6/40/htm. Ovarian cancer is one of the most fatal cancers for women because it lacks early diagnosis and has a high risk of reoccurrence. Current chemotherapy is with extensive side effects and doesn't work on certain types of ovarian cancers. In this study, a natural compound called trichodermin was found to preferentially inhibit the proliferation of cisplatin-sensitive and cisplatin-resistant ovarian cancer cells but not normal cells. Trichodermin decreased the proliferation by inducing the G0/G1 cell cycle arrest. C-Myc, a transcription factor often found overexpressed in cancerous tissues, was the key target of trichodermin. Trichodermin suppressed the expression of c-Myc and thereby reduced the expressions of several c-Myc downstream proteins, many of which were involved in maintaining the progression of the cell cycle. The anti-ovarian cancer effect of trichodermin was demonstrated in BALB/c nude mice. The average tumor size was smaller in the trichodermin group compared with that in the vehicle group. Besides, no obvious side effects were observed in mice during the experiment period. These findings suggested that trichodermin has the potential to contribute to the treatment of ovarian cancer.
